# Perceived etiology of foodborne illness among public health personnel.

**DOI:** 10.3201/eid0705.010525

**Published:** 2001

**Authors:** T F Jones, D E Gerber

**Affiliations:** Tennessee Department of Health, Communicable and Environmental Diseases Services, Nashville 37247, USA. tjones4@mail.state.tn.us

## Abstract

Few data exist about perceptions regarding the etiology of foodborne illness. Among public health staff throughout Tennessee, the three pathogens most commonly believed to cause foodborne illness in the United States actually account for only 12% of disease. Fewer than 3% of respondents correctly identified the leading cause of foodborne illness.


### Perceived Etiology of Foodborne Illness Among Public Health Personnel

**Timothy F. Jones and Diane Eigsti Gerber**
Tennessee Department of Health, Nashville, Tennessee, USA

Few data exist about perceptions regarding the etiology of foodborne illness. Among public health staff throughout Tennessee, the three pathogens most commonly believed to cause foodborne illness in the United States actually account for only 12% of disease. Fewer than 3% of respondents correctly identified the leading cause of foodborne illness.

In the United States, foodborne infections cause approximately 76 million illnesses each year, accounting for 325,000 hospitalizations and 5,000 deaths (1). Foodborne illness has been estimated to cost as much as $23 billion annually in this country (2). The consequences of such illness can range from transient discomfort to meningitis, congenital malformation, and death (3). Changes in eating habits and food preparation behaviors, globalization of the food supply, aging of the population, and other risk factors may be leading to increasing rates of illness (4,5).

Public health and infection control personnel are frequently involved in the identification, investigation, and intervention of foodborne illness outbreaks. In 68% of reported foodborne outbreaks in the United States, the pathogenic cause is not identified (6). An understanding of likely etiology is important for ensuring appropriate management of illness. There are few published data on the knowledge or perceptions of public health personnel regarding the common causes of foodborne disease.

### Methods

During April and May 2000, epidemiologists, laboratory staff, and environmentalists from the Tennessee Department of Health presented a series of lectures to public health personnel throughout the state to review the process of investigating foodborne illness outbreaks. Participants included epidemiologists, public health nurses, laboratory staff, and environmentalists.

Before each session, participants were asked the following question: "What are the three most common pathogens causing foodborne illness in the United States?" Verbal instructions included clarification that the question referred to which pathogens were numerically the most frequent causes of illness. Participants ranked their top three answers in writing and submitted them to the course director. Responses were anonymous, although the job category of each respondent was collected. Data were entered and analyzed by using EpiInfo software (7).

Address for correspondence: Timothy F. Jones, Tennessee Department of Health, FoodNet Program, Communicable and Environmental Disease Services, 425 5th Avenue, 4th Floor, Cordell Hull Building, Nashville, TN 37247, USA; fax: 615-741-3857; e-mail: tjones4@mail.state.tn.us

### Results

Of 553 attendees, 388 (70%) participants responded to the survey. Respondents included 128 environmentalists, 233 public health nurses, 11 health department physicians, 4 laboratorians, and 12 persons in other positions in the health department. The proportion of participants was representative of the proportion of responders to foodborne illness within the health department.

Ninety percent of persons listed *Salmonella* among the top three most common causes of foodborne illness in the

Table. Percentage of respondents identifying each pathogen as among the top three causes of foodborne illness, and estimated percentage of foodborne illnesses in the United States actually caused by those pathogens

| Pathogen | Percentage of respondents listing it among top three causes | Est. percentage of foodborne illness in USA caused by pathogen (1) |
|---|---|---|
| *Salmonella* | 90 | 9.7 |
| *Escherichia coli* | 56 | 1.3 |
| *Staphylococcus* | 36 | 1.3 |
| *Shigella* | 32 | 0.6 |
| *Campylobacter* | 18 | 14.2 |
| *Listeria* | 16 | <0.1 |
| *Hepatitis A virus* | 8 | <0.1 |
| *Clostridium perfringens* | 8 | 1.8 |
| *Norwalk-like virus* | 5 | 66.7 |
| Viruses[a] | 4 | 67.2 |
| *Giardia lamblia* | 3 | 1.4 |
| *Streptococcus* | 2 | 0.4 |

[a]Respondents who wrote in "viruses" only; does not include those who specified *Norwalk-like virus*.
Est = estimated.

United States; 56% listed *Escherichia coli*; 36% cited *Staphylococcus*; and 32% *Shigella* (Table). Other commonly cited causes of foodborne illness included *Campylobacter, Listeria, Hepatitis A virus*, and *Clostridium*.

Only 5% of respondents listed *Norwalk-like virus* (NLV) among the three most common causes of foodborne illness, and an additional 4% noted “viruses” more generically. Only 4% of respondents listed NLV or viruses as the most common source of foodborne illness.

Results did not vary significantly by job category of the respondents. Public health nurses, environmentalists, and physicians, for example, all listed *Salmonella, E. coli, Staphylococcus*, and *Shigella* as the most common causes of foodborne illness. Persons from all job categories were represented among the 9% of respondents who listed viruses or NLV among the top three causes. No job category was statistically more likely to identify viruses as a common etiology, and in no group was NLV among the five most commonly listed pathogens.

### Conclusions

The four pathogens most commonly believed by the survey respondents to be among the major causes of foodborne illness (*Salmonella, E. coli, Staphylococcus*, and *Shigella*) are actually estimated to account for <13% cumulatively of all foodborne disease in the United States (Table). Recent estimates suggest that the most common causes of foodborne illness in the United States, in decreasing order of frequency, are NLV, *Campylobacter, Salmonella, Clostridium perfringens*, and *Giardia* (1). Only 5% of respondents listed NLV among the three most common causes of foodborne illness; this agent is estimated to cause 67% of all foodborne disease in the United States (1). In contrast, *Listeria*, which causes <0.1% of foodborne illness in the country, was believed by 15% of respondents to be among the three most common causes of disease.

The response to a suspected foodborne illness may differ, depending on the likely etiology. Basic methods of case-finding, hypothesis-generating, and investigating exposure histories do not necessarily require knowledge of the frequency of possible pathogens. Other issues, such as stool collection and testing techniques, treatment and follow-up, and preventive recommendations may differ greatly depending on a particular pathogen. If the personnel commonly responsible for recognizing, reporting, and intervening in foodborne illness are unfamiliar with common pathogenic causes of such illnesses, the appropriateness of their responses may be compromised.

Reasons for the discrepancy between perceived and actual etiologies of foodborne illness are unknown. While estimates that two-thirds of foodborne illnesses are caused by caliciviruses may be debated, perceptions of study respondents reflect neither national estimates nor recent experience in Tennessee. Some pathogens incorrectly believed to be common causes of foodborne illness, such as *E. coli* and *Listeria*, cause relatively severe disease, which often generates substantial media attention. Highly publicized outbreaks and severe cases may disproportionately affect perception of a pathogen’s incidence. Such factors might be expected to influence public perception more than that of health-care workers, although this study suggests otherwise. There is no evidence that the public health personnel we surveyed have a substantially different understanding than health-care workers elsewhere. Studies on factors that affect both academic knowledge of the causes of foodborne illness, as well as factors such as severity and risk (which likely strongly influence perception of their relative importance), would be of value.

While it is true that the etiology is not identified in a large proportion of foodborne illnesses, lack of knowledge on the part of public health personnel is only one barrier to improving this situation. Lack of resources, competing priorities, the health-seeking behaviors of ill persons, and the activities of clinical and laboratory providers all have important effects on the response to suspected foodborne illness. Despite that, this study suggests that public health personnel on the front lines in responding to foodborne illness have incorrect perceptions of its causes. If this substantial public health threat is to be effectively addressed, appropriately educating the persons relied upon to address the problem is necessary.

### Acknowledgments

The authors thank Allen Craig for assistance in data collection and William Schaffner for his review of the manuscript.

Dr. Jones is an epidemiologist and director of Tennessee’s Emerging Infections Program’s Foodborne Illness Active Surveillance Network (FoodNet). He was an officer in the Center for Disease Control and Prevention’s Epidemic Intelligence Service and currently focuses on foodborne illness and tuberculosis in the Tennessee Department of Health.

Ms. Gerber is a public health nurse and educator, currently working as a surveillance officer and FoodNet coordinator in the Tennessee Department of Health.

### References

1. Mead PS, Slutsker L, Dietz V, McCaig LF, Bresee JS, Shapiro C, et al. Food-related illness and death in the United States. Emerg Infect Dis 1999;5:607-17.
2. Hedberg CW, MacDonald KL, Shapiro C. Changing epidemiology of foodborne disease: a Minnesota perspective. Clin Infect Dis 1994;18:671S-82S.
3. Altekruse SF, Cohen ML, Swerdlow DL. Emerging foodborne diseases. Emerg Infect Dis 1997;3:285-93.
4. Bender JB, Smith KE, Hedberg C, Osterholm MT. Food-borne disease in the 21st century. What challenges await us? Postgrad Med 1999;106:109-19.
5. Yang S, Leff MG, McTague D, Horvath KA, Jackson-Thompson J, Murayi T, et al. Multistate surveillance for food-handling, preparation, and consumption behaviors associated with foodborne diseases: 1995 and 1996 BRFSS food-safety questions. MMWR Morb Mortal Wkly Rep 1998;47:33-41.
6. Centers for Disease Control and Prevention. Surveillance for foodborne-disease outbreaks–United States, 1993-1997. MMWR Morb Mortal Wkly Rep 2000;49(No. SS-1):4.
7. Dean AD, Dean JQ, Coulombier D, Brendel KA, Smith DC, Burton AH, et al. EpiInfo. Version 6: a word processing, database and statistics program for epidemiology on microcomputers. Atlanta, GA: Centers for Disease Control and Prevention; 1994.

## References

[PDF_00196] Altekruse S. F., Cohen M. L., Swerdlow D. L. (1997). Emerging foodborne diseases.. Emerg Infect Dis.

[PDF_00198] Bender J. B., Smith K. E., Hedberg C., Osterholm M. T. (1999). Food-borne disease in the 21st century. What challenges await us?. Postgrad Med.

[PDF_00190] Mead P. S., Slutsker L., Dietz V., McCaig L. F., Bresee J. S., Shapiro C., Griffin P. M., Tauxe R. V. (1999). Food-related illness and death in the United States.. Emerg Infect Dis.

